# 

**Published:** 1882-06

**Authors:** 


### CONTENTS.

### *MISCELLANEOUS.*

| | PAGE. |
|---|---|
| Which? Editorial, | 241 |
| Misrepresentations. Ad. Lippe, M. D., Philadelphia, | 243 |
| Hering's Analytical Therapeutics. P. P. Wells, M. D., | 247 |
| The Study and Practice of Homœopathy. D. Wilson, M. D., | 250 |
| Central New York Homœopathic Society. C. P. Jennings, Sec'y, | 259 |
| Ague and Quinine. E. J. L., | 263 |
| Norton's Ophthalmic Therapeutics. Chas. Deady, M. D., | 268 |
| Notes on Genito-Urinary Therapeutics. E. J. L., | 270 |
| Index of Cough Symptoms. Drs. Clark and Lee, | 278 |

### *CLINICAL BUREAU:*

| | |
|---|---|
| Ledum in Cutaneous Affections. L. B. Wells, M. D., Utica, N. Y., | 272 |
| High Potencies in Acute as well as Chronic Diseases. A. J. Brewster, M. D., Syracuse, N. Y., | 273 |
| A Case from Practice. E. P. Hussey, M. D., Buffalo, N. Y., | 276 |
| Meeting of the International Hahnemannian Association, | 277 |

### *BOOKS:*

| | |
|---|---|
| Angell. Diseases of the Eye. Bœricke & Tafel, | 286 |
| Butler. Electricity in Surgery. Bœricke & Tafel, | 287 |
| Cushing. Leucorrhœa, etc. Mudge & Son, | 287 |
| Smith's New Label-holder. G. W. Smith, | 287 |

### *NOTES AND NOTICES:*

| | |
|---|---|
| Removals—Long Branch—Worse Yet—The Kind Virginia Wants—A Farce and a Tragedy. | 288 |

